# An Unexpected Challenge: Marked Small Bowel Obstruction Arising From a Poorly Differentiated Metastatic Mucinous Adenocarcinoma of the Distal Ileum in a Risk-Free Healthy Patient

**DOI:** 10.7759/cureus.61528

**Published:** 2024-06-02

**Authors:** Jennie An, Barbara Fontenelle, Riddhi Chaudhari, Rob Mulloy, Sofiane El Djouzi

**Affiliations:** 1 Medicine, St. James School of Medicine, Park Ridge, USA; 2 General Surgery, AdventHealth, Bolingbrook, USA

**Keywords:** no risk factors, adenocarcinoma of the ileum, partial ileal resection, small bowel neoplasm, ileal adenocarcinoma, small bowel adenocarcinoma

## Abstract

Adenocarcinoma, while constituting the predominant variant among small bowel cancers, is a component of the broader category of primary small bowel malignancies, which are notably infrequent in occurrence. The diagnosis of such malignancies is often markedly delayed, a consequence of their insidious onset and the nonspecific nature of the abdominal symptoms presented.

A 69-year-old Caucasian male presented to the emergency department manifesting acute, sharp, and colicky abdominal pain accompanied by a single episode of vomiting, all developing over one day. His medical history was notable for gastroesophageal reflux disease (GERD) and regionally confined prostate adenocarcinoma, which was under meticulous surveillance by the urological team. The patient's lifestyle was characterized by abstention from alcohol and tobacco, adherence to a nutritious diet, and a commitment to regular physical activity. Subsequent examination and surgical excision of an abnormal mass, as delineated on computed tomography (CT), culminated in the diagnosis of a stage IV, poorly differentiated adenocarcinoma.

We have reported this case to spark research regarding early diagnostic techniques for small bowel adenocarcinoma (SBA). In this case, a healthy individual presented with vague abdominal pain and a single episode of vomiting. Diagnosis required the surgical resection of the tumor, where metastasis was also visualized. Due to the rare nature of SBA, we believe different diagnostic measures and adjuvant therapy should be researched for earlier diagnosis and subsequently better patient outcomes.

## Introduction

Adenocarcinoma of the ileum is a rare malignant tumor accounting for only 10% of all small bowel cancers [1-4]. It often presents late with the onset of nonspecific symptoms, such as vague abdominal pain and nausea, prior to exhibiting more specific signs such as small bowel obstruction. Small bowel obstruction can present with vomiting and inability to pass flatus [3].

The common associations of small bowel adenocarcinoma (SBA) are chronic inflammatory diseases such as inflammatory bowel disease like Crohn's disease, and celiac disease. SBA is also associated with sporadic mismatch repair (MMR) deficiency leading to subsequent microsatellite instability (MSI) and familial adenomatous polyposis (FAP) [4]. More so, a significant cohort showed that predisposing lifestyle factors, in addition to sporadic or genetic causes, can be found in 20% of cases of SBA [1]. Lifestyle and environmental factors, such as smoking, highly processed diets, and sedentary lifestyles, have also been hypothesized to play a role in the occurrence of adenocarcinoma of the ileum [2,3]. Regardless, the majority of cases reported for adenocarcinoma of the ileum are associated with a risk factor as listed above. The case becomes challenging when an individual without any known factors, including a genetic or sporadic mutation, a family history of cancer, inflammatory bowel disease, and a healthy lifestyle, develops obstructive high-grade adenocarcinoma of the small bowel.

In this report, we present the case of a 69-year-old male presenting with mild signs of small bowel obstruction with no known risk factors. Due to the rarity of the disease, insidious onset, and vague non-specific abdominal symptoms, this case report highlights the need for careful review of vague abdominal symptoms to improve patient diagnostic and treatment outcomes [5].

## Case presentation

Our patient, a 69-year-old Caucasian male, a retired music teacher, presented to the emergency department with a one-day history of sharp and colicky upper abdominal pain with nausea and one episode of vomiting. Absolutely no other symptoms were noted by the patient and no other signs were noticed by the surgeon prior to surgery. Vitals showed elevated blood pressure yet the patient was hemodynamically stable. His BMI was 23.3. He denied any other abdominal history and a family history of malignancy. This was confirmed by genetic testing, immunohistochemistry, and blood tests. His past medical history consisted of gastroesophageal reflux disease (GERD) and localized prostate adenocarcinoma diagnosed a year ago, which was being meticulously monitored by the urological team. GERD was confirmed via endoscopy and no dysplasia was present. Pantoprazole was being used for the treatment of GERD and is well managed. The patient does not exhibit signs of prostate cancer such as urinary issues (increased frequency, incontinence), blood in urine, or bone pains. The fecal blood test was negative for occult blood. The patient was abstinent from smoking and drinking, consumed a healthy diet, and went to the gym three to five times per week. The latest colonoscopy was done five years prior, and no abnormalities were seen.

**Figure 1:**
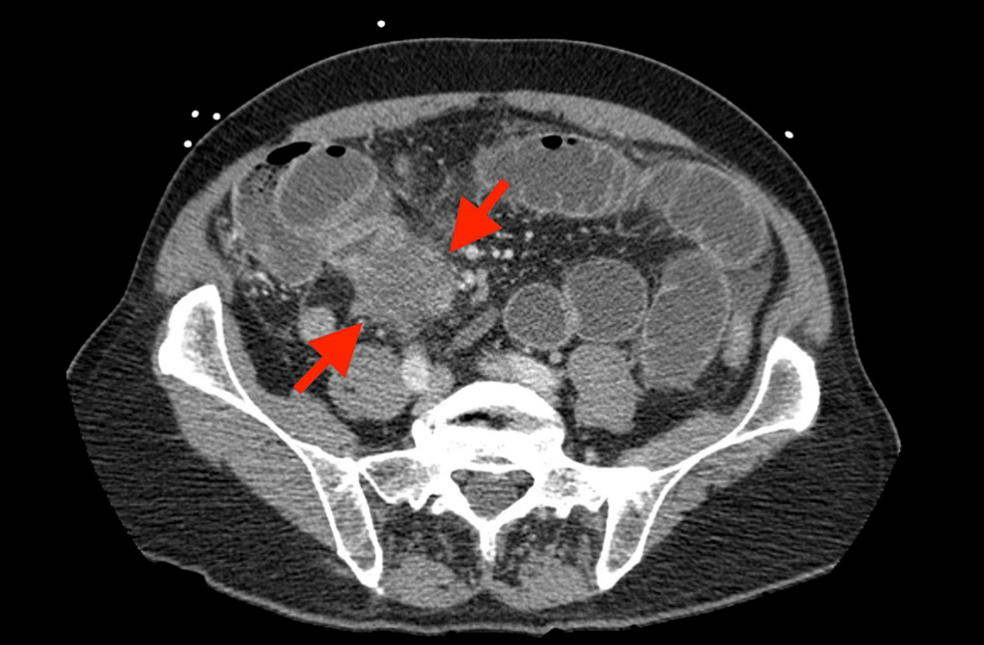
Axial CT of the abdomen shows a mass in the distal ileal (approximately 5 cm in diameter) in the right lower quadrant CT: computed tomography scan

Initial lab workup in the emergency department showed mild anemia, leukocytosis, lymphopenia, and significant neutrophilia. A computerized tomography (CT) scan of the abdomen and pelvis (Figure 1) revealed a 5 cm small bowel mass in the right lower quadrant, causing obstruction. Concerns for metastatic lesions throughout the peritoneum and hypoechoic lesions within the liver were noted.

**Figure 2:**
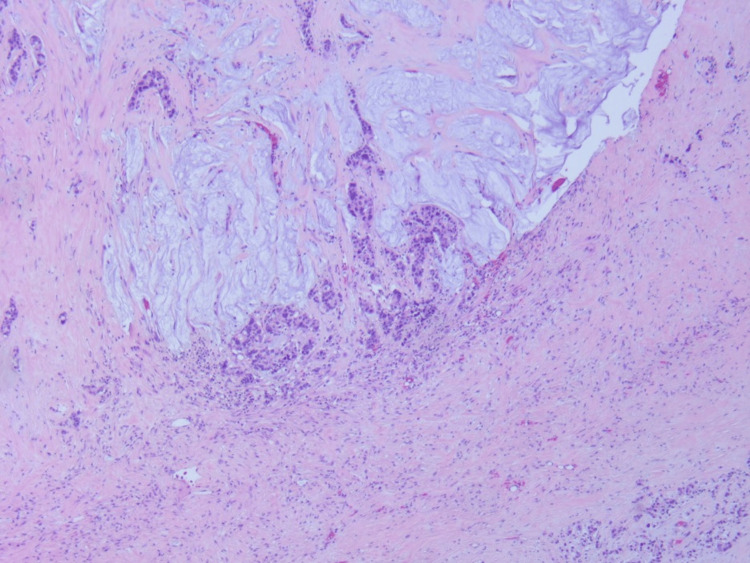
Hematoxylin and eosin staining illustrating the positive (cancerous) and negative (noncancerous) margins

**Figure 3:**
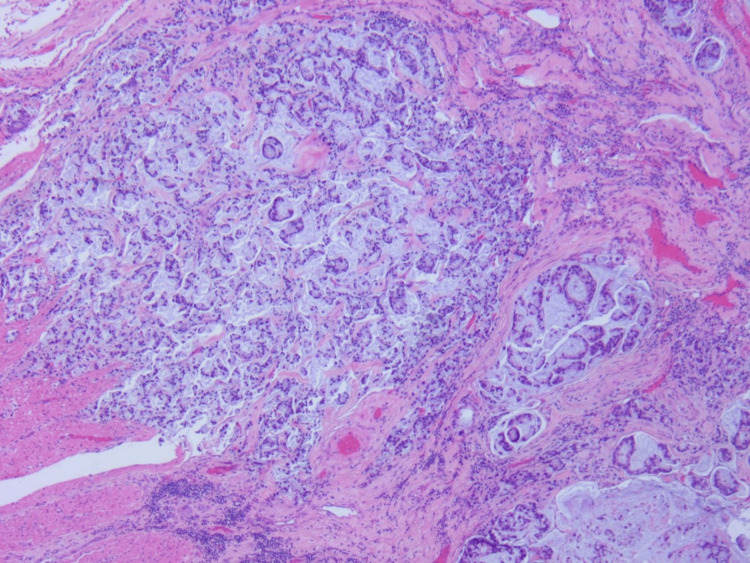
Hematoxylin and eosin staining, showing poorly differentiated adenocarcinoma with mucinous and signet-ring cell features

**Figure 4:**
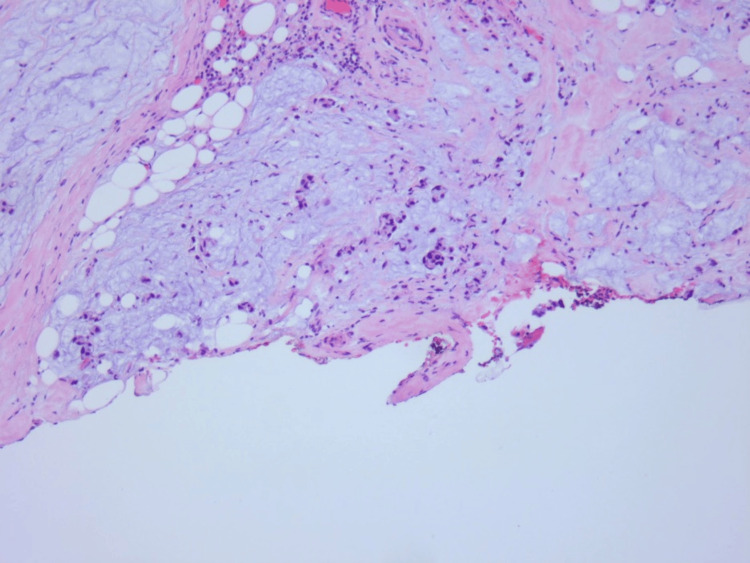
Mesenteric margin showing the poorly differentiated signet-ring cell features

The ileal tubular mass, as shown in Figure 2, and the associated small bowel were resected and anastomosed via robotic surgery. Two peritoneal tumoral deposits were taken. Biopsy of the mass and the deposits demonstrated G3, poorly differentiated adenocarcinoma with mucinous signet ring cell features as shown in Figures 3-4. Notably, out of 10 examined lymph nodes, 3 exhibited tumor infiltration. Immunohistochemistry did not show MMR mutations and DNA analysis was not notable for APC mutations.

Following a week-long postoperative recovery, the patient was discharged and scheduled to commence chemotherapy as part of his treatment plan.

## Discussion

This rare malignancy often presents with an indolent course of vague abdominal pain. Our patient presented with a one-day history of sharp and colicky pain without any significant risk factors. Despite being rare, primary small bowel malignancies are associated with risk factors, such as diet, chronic inflammatory bowel disease, maladaptive immune disorders, and hereditary intestinal disorders, which predispose individuals to malignancies. In the case of obstructive malignancy, the primary treatment is the removal of the mass and further consultation with oncology. Despite researching previous case reports, it was rare to find a risk-free healthy patient with poorly differentiated metastatic adenocarcinoma of the small bowel.

Epidemiology

The incidence of small bowel adenocarcinoma has doubled over the past 40 years and is attributed to the lifestyle changes of the developed world such as physical inactivity, processed or unhealthy diets, and occupation [6]. Among these risk factors, Ginzburg first discussed that small bowel adenocarcinoma is a rare complication of Crohn's disease and is often found in male patients [6]. Despite the small bowel making up about 75% of the intestinal tract, cancer of this region accounts for only 5% of all gastrointestinal malignancies [1]. Epidemiologically speaking, SBAs typically occur in elderly male patients between the ages of 50 and 70 years old [7-10]. Although only making up 1-3% of all GI malignancies, the majority of SBA occurrence has been associated with inflammatory bowel disease and hereditary cancer syndromes, such as Crohn's disease, and familial adenomatous polyposis (FAP) or hereditary nonpolyposis colorectal cancer (HNPCC), respectively [3]. More so, neuroendocrine tumors have become the most common type of tumor to occur in the jejunum (25-29%) and ileum (10-13%) while duodenal involvement is the most commonly involved (60%). It has been theorized that the proximity to the ampulla of Vater, due to various metabolites and bile, could play a role in the pathophysiology of small bowel adenocarcinoma [1]. Unfortunately, the 5-year survival rate for small bowel adenocarcinoma remains low, around 35%, due to inadequate sample size and research surrounding prevention and treatment. The 5-year survival rate is even lower in patients with spread to regional lymph nodes (35%) and distant metastases (27%) [7].

Paradoxically, SBAs are rare despite it being the predominant location of absorption. Some hypotheses attribute the occurrence of SBAs to the high caliber and rapid transit of the small bowel, whereas others think it could be due to higher levels of immunoglobulin A (IgA) [8]. In the absence of predisposing disease, other risk factors, such as consumption of highly processed diets or alcohol, smoking, and sedentary lifestyles are often present. In our case, the patient did not have any risk factors noted in past research. More so, the patient exercised regularly and ate a healthy diet. 

Current diagnostic methods

The multitude of imaging techniques have not been diagnostic for SBA. These imaging techniques include plain abdominal X-rays, upper gastrointestinal series with small bowel follow-through (barium), enteroclysis, CT, CT enterography, magnetic resonance (MR) enterography, positron emission tomography-CT (PET-CT), and small bowel video capsule endoscopy (VCE) [11]. Although each technique has its advantages, not one allows for a definitive diagnosis of SBA. X-rays and upper GI series can show the neoplasm or obstruction, but neither allows for proper staging and smaller tumors could be missed. CT scans, which were conducted on our patient, are commonly used to detect distant metastasis and lymphatic spread. Currently, CT enterography and MR enterography are the preferred methods of diagnosis since they also allow for the staging of the cancer. These techniques allow for the visualization of the abnormal mucosa present in the small bowel wall [12].

Treatment

The initial step of treatment of SBA is surgical resection. However, even with the resection, the prognosis is still poor [5]. This is largely due to the lack of adjuvant therapy. The rare nature of SBA does not allow for clinical trials that test different treatments and management [11]. Adjuvant chemotherapy did not increase the survival of patients with nodal metastasis, but a slight increase in survival in patients with stage I/II cancer and a significant increase in patients with stage III cancer [13]. Chemoradiation as adjuvant therapy is only used in duodenal adenocarcinomas due to their high rate of local relapse.

Despite advances in understanding the disease and its treatment modalities, the therapeutic landscape for SBA remains challenging and often inadequate [10,14-17]. Continued efforts to explore novel adjuvant therapies and refine treatment protocols are essential to improve outcomes for patients afflicted with this rare and aggressive malignancy.

Literature review

Etiology and Risk Factors

The etiology of small bowel adenocarcinoma has a multifactorial influence by a complex mix of genetic, environmental, and lifestyle factors [18]. Chronic inflammatory conditions like Crohn's disease and celiac disease significantly raise the chances of SBA occurrence, with research indicating that these conditions can lead to changes in the small intestine over time [19]. Additionally, genetic predispositions, such as adenomatous polyposis (FAP), are closely associated with a higher risk of SBA, suggesting a strong hereditary element in the disease’s onset [9]. Lifestyle choices like diets rich in processed foods and red meat along with a lack of physical activity have been linked to an increased risk as well although the exact mechanisms are still being studied. Environmental factors, such as radiation and specific chemicals, may also play a role in the risk factors, to a lesser degree. Despite these known risk factors, a significant number of SBA cases occur in individuals without any identified predispositions highlighting the importance of research to uncover additional causes that could contribute to the disease.

It is reported that 10% of colonic polyps become adenocarcinoma within 10-20 years [18]. Often, these polyps are found early, monitored, or removed, preventing the progression to adenocarcinoma. SBA is not subject to the same screening due to the rare nature of the disease and lack of research. Current studies have attributed the occurrence of SBA in part to lifestyle, diet, and genetics.

Current Case Reports

In addition to most cases of SBA being diagnosed at a late stage due to its rare and indolent nature, its non-specific presentation also contributes to frequent misdiagnoses, which in turn may lead to delayed treatment and consequent poor outcomes for the patient.

D. Lim and colleagues conducted a case series and review, focusing on two patients who exhibited the typical symptoms of SBA. These symptoms included non-radiating abdominal pain, absence of hematochezia, and recent weight loss [20]. The first patient had a significant smoking history of 45 pack-years and a family history of both bowel and breast cancer. In contrast, the second patient lacked both a smoking history and any family history of cancer. Both patients were mistakenly diagnosed with Crohn's disease based on imaging. Lim found that the MRI and CT scan findings of SBA often mimic inflammatory bowel disease. In a similar case study by M. Bouali and colleagues [3], a patient was incorrectly diagnosed and treated for celiac disease for one year. This study emphasized the challenges of distinguishing between inflammatory diseases from locally invasive adenocarcinoma based on imaging alone.

Furthermore, it is important to highlight that patients frequently receive immunosuppressants and steroids to manage their misdiagnosed disease. While these medications can initially alleviate symptoms [19,20], they often delay the accurate diagnosis of SBA and appropriate treatment.

## Conclusions

This case reveals a poorly differentiated metastatic adenocarcinoma of the large distal ileum, which manifested with an indolent course followed by nonspecific and specific symptoms. Despite extensive research elaborating on the risk factors and pathogenesis of SBA, little attention has been devoted to its occurrence in seemingly healthy, risk-free individuals. Our patient, in particular, perplexed us as we searched for risk factors and potential reasons for the disease, yet were left without answers. Palliative chemotherapy and immunotherapy, in addition to surgery, are the current treatments for SBA, yet the prognosis for such malignancies remains penurious. Current imaging techniques fail to detect luminal malignancies at early stages, depriving patients of early treatment and bettering prognosis. We present this report to urge the stipulation for research pertaining to new risk factors yet discovered, early and/or non-invasive diagnostic techniques, and efficacious treatment to yield a better prognosis in SBA patients.
